# Cm-p5 Peptide Dimers Inhibit Biofilms of *Candida albicans* Clinical Isolates, *C. parapsilosis* and Fluconazole-Resistant Mutants of *C. auris*

**DOI:** 10.3390/ijms24129788

**Published:** 2023-06-06

**Authors:** Valerie Amann, Ann-Kathrin Kissmann, Vanessa Mildenberger, Imke Krebs, Julio A. Perez-Erviti, Ernesto M. Martell-Huguet, Anselmo J. Otero-Gonzalez, Fidel Morales-Vicente, Gina P. Rodríguez-Castaño, Carolina Firacative, Armando Rodríguez, Ludger Ständker, Tanja Weil, Barbara Spellerberg, Steffen Stenger, Frank Rosenau

**Affiliations:** 1 Institute of Pharmaceutical Biotechnology, Ulm University, Albert-Einstein-Allee 11, 89081 Ulm, Germany; valerie.amann@uni-ulm.de (V.A.); ann-kathrin.kissmann@uni-ulm.de (A.-K.K.); vanessa.mildenberger@uni-ulm.de (V.M.); imke.krebs@uni-ulm.de (I.K.); 2 Max Planck Institute for Polymer Research Mainz, Ackermannweg 10, 55128 Mainz, Germany; weil@mpip-mainz.mpg.de; 3 Center for Protein Studies, Faculty of Biology, University of Havana, 25 Str. and I Str., La Habana 10400, Cuba; japerviti@gmail.com (J.A.P.-E.); nestmartell@gmail.com (E.M.M.-H.); aotero@fbio.uh.cu (A.J.O.-G.); 4 Core Facility for Functional Peptidomics, Ulm Peptide Pharmaceuticals (U-PEP), Faculty of Medicine, Ulm University, 89081 Ulm, Germany; armando.rodriguez-alfonso@uni-ulm.de (A.R.); ludger.staendker@uni-ulm.de (L.S.); 5 Synthetic Peptides Group, Center for Genetic Engineering and Biotechnology, La Habana 10600, Cuba; femvicente@gmail.com; 6 Vidarium Nutrition, Health and Wellness Research Center, Grupo Nutresa, Calle 8 sur #50-67, Medellín 050023, Colombia; gprodriguez@serviciosnutresa.com; 7 Studies in Translational Microbiology and Emerging Diseases (MICROS) Research Group, School of Medicine and Health Sciences, Universidad de Rosario, Bogota 111221, Colombia; cfiracative@gmail.com; 8 Core Unit of Mass Spectrometry and Proteomics, Faculty of Medicine, Ulm University, 89081 Ulm, Germany; 9 Institute for Medical Microbiology and Hygiene, University Hospital Ulm, 89081 Ulm, Germany; barbara.spellerberg@uniklinik-ulm.de (B.S.); steffen.stenger@uniklinik-ulm.de (S.S.)

**Keywords:** antimicrobial peptides, biofilm inhibition, *Candida* species, clinical isolates, resistances

## Abstract

Antimicrobial peptides (AMPs) represent a promising class of therapeutic biomolecules that show antimicrobial activity against a broad range of microorganisms, including life-threatening pathogens. In contrast to classic AMPs with membrane-disrupting activities, new peptides with a specific anti-biofilm effect are gaining in importance since biofilms could be the most important way of life, especially for pathogens, as the interaction with host tissues is crucial for the full development of their virulence in the event of infection. Therefore, in a previous study, two synthetic dimeric derivatives (parallel Dimer 1 and antiparallel Dimer 2) of the AMP Cm-p5 showed specific inhibition of the formation of *Candida auris* biofilms. Here we show that these derivatives are also dose-dependently effective against de novo biofilms that are formed by the widespread pathogenic yeasts *C. albicans* and *C. parapsilosis*. Moreover, the activity of the peptides was demonstrated even against two fluconazole-resistant strains of *C. auris*.

## 1. Introduction

**Figure 1:**
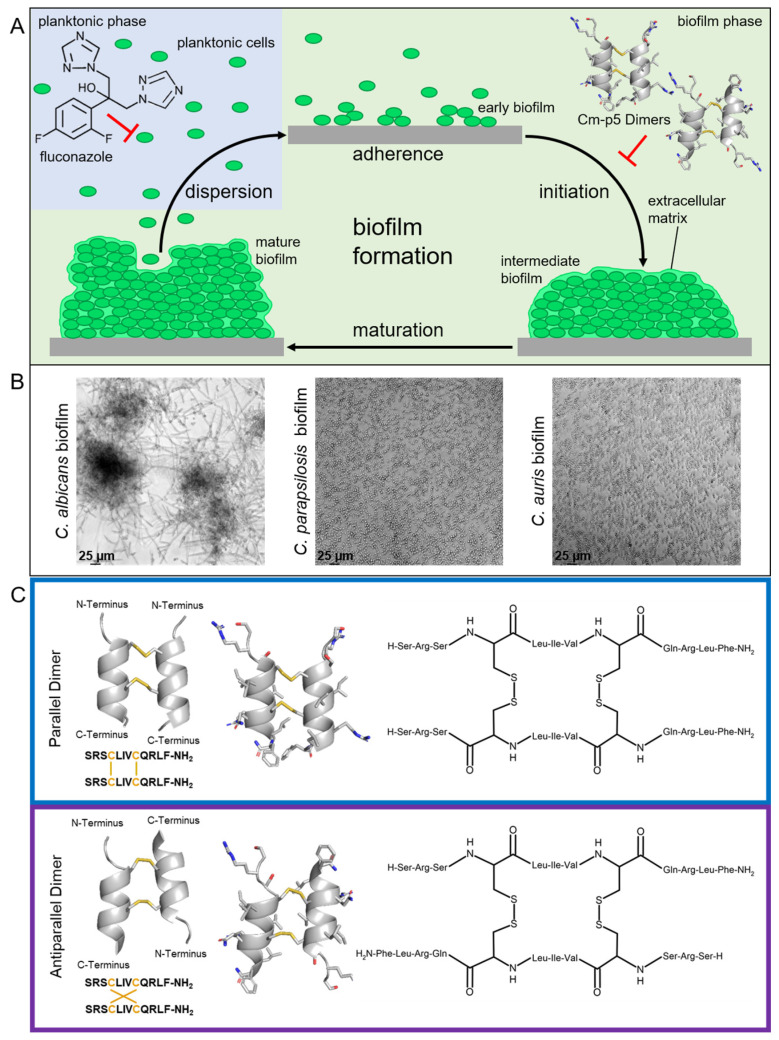
(**A**) Overview of a typical *Candida* biofilm development. Classification of biofilm formation process is divided into four major steps: (I) The adherence of planktonic yeast cells to the future biofilm substratum; (II) the initiation of biofilm formation by aggregation and proliferation of cells as well as the production of an extracellular matrix (intermediate biofilm); (III) the development of a species-specific mature biofilm; and (IV) the dispersion of planktonic cells from the mature biofilm, which delivers *Candida* seeds for the next generation of biofilms. Antifungal activity of fluconazole on yeast cells in the planktonic phase and anti-biofilm effect of Cm-p5 Dimers. (**B**) Microscopy of biofilms formed by *C. albicans*, *C. parapsilosis,* and *C. auris* after 24 h growth without agitation on polystyrene in RPMI-1640 medium. Microscopic analysis under transmitted light at ×20 magnitude using a Leica DMi8 coded (Leica Microsystems CMS GmbH, Wetzlar, Germany). (**C**) Modeled 3D structures of parallel Dimer 1 and antiparallel Dimer 2 using the QUARK and SwissModel servers and the corresponding amino acid sequences. Disulfide bonds are illustrated in orange, side chain carbon, oxygen, and nitrogen atoms are displayed in grey, red, and blue, respectively [11].

Fungi, nowadays termed the “hidden killers”, cause roughly 1.5 million lethal infections worldwide, which are as many as HIV and even more than malaria [1]. The majority of these severe fungal infections are attributed to *Candida* species, as candidiasis represents the fourth leading cause of nosocomial infections with high mortality rates in systemic courses ranging from 15–35% [2,3]. The most frequently isolated species and worldwide most prevalent fungal pathogen is the yeast *Candida albicans* [4,5]. However, non-*albicans* Candida (NAC) species such as *C. parapsilosis* or *C. auris* now account for a significant proportion of clinical isolates, and their observation rate has increased dramatically over the past 15 years [6]. In particular, 65% of nosocomial infections, or up to over 80% of all microbial infections, in general, are associated with the formation of biofilms, as estimated by the National Institute of Health (NIH) [7]. Biofilms are organized consortia of cells, and these microbial communities are usually enclosed by an extracellular matrix formed after cells adhere to their substratum (Figure 1A,B) [8,9]. Due to the formation of a biofilm, yeast cells enhance both their morbidity and mortality, as well as their physical stability to antifungal drugs, which complicates the treatment of fungal infections caused by these *Candida* species [10]. After the maturation of a species-specific biofilm, planktonic cells are then released from the consortium and serve as seeds for the next generation of biofilms.

Fluconazole is a triazole antifungal and one of the most commonly prescribed agents for infections with *Candida* species [12]. In general, azoles inhibit the cytochrome P450 enzyme 14α-demethylase, encoded by *ERG11*, in the biosynthesis pathway of ergosterol, which is an essential compound of the cell membranes of fungi [13]. There, fluconazole prevents the conversion of lanosterol to ergosterol, as the free nitrogen of the azole ring can bind to an iron atom within the heme group of the 14α-demethylase. Consequently, the activation of oxygen is prevented, and thus the demethylation of lanosterol inhibits the progress of biosynthesis [14]. The inhibition of ergosterol formation leads to the accumulation of methylated sterols in the cellular membranes of the fungi, resulting in arrested cell growth [15].

A low incidence of resistance against fluconazole is observed in *C. albicans* clinical isolates, with rates ranging from 0.5 to 2%, but the rates exhibited by *C. parapsilosis* are slightly higher at 2.5–6% [16,17]. However, clinical isolates of the emerging pathogenic yeast *C. auris* showed an alarming rate of resistance to fluconazole as high as 93% [18,19]. The level of fluconazole resistance in *C. auris* isolates is of particular concern as it remains the most widely prescribed antifungal and many of the *C. auris* outbreaks have occurred in environments with limited clinical resources [12,20,21]. The incidence of this yeast in Europe is low compared to outbreaks in Asia, South Africa, or the USA. Particularly in Germany, *C. auris* affected seven patients between January 2015 and May 2017, six of whom had previously had contact with foreign healthcare providers or were hospitalized abroad [22,23]. Moreover, two cases of *C. auris* infections without prior contact with foreign healthcare were recently reported in Germany, and they emerged even in an Italian hospital in connection with the COVID-19 pandemic [24,25]. While the spread of fluconazole resistance in clinical *C. auris* isolates significantly limits the therapeutic options for infections, the molecular mechanisms underlying this resistance remain not fully clear but have been discussed previously. For instance, several mutations were associated with an increased expression of *ERG11* as well as an increased ergosterol production [26]. There, the elevated production of the azole target molecule decreases the efficacy of fluconazole and thus results in resistance [27]. In addition to mechanisms involving the pathway of ergosterol biosynthesis, the efflux of antifungals via transport proteins plays a crucial role in the formation of drug resistance. In *C*. *albicans,* two main classes of transport proteins, the major facilitator superfamily (MFS) class and the ATP-binding cassette (ABC) family, lead to a failure of intracellular drug accumulation and hence to azole resistance [28,29]. Furthermore, these efflux pumps are even upregulated in the biofilms of multi-resistant *C. auris,* and a third of the clinical isolates exhibit increased MIC levels for drugs from two or more different classes of antifungal drugs [30,31,32]. Emerging resistance to all classical antifungals has provoked the urgent need to overcome these limitations through alternative mechanisms of action. Therefore, a promising class of therapeutic molecules is antimicrobial peptides (AMPs) [33,34]. AMPs represent a class of biomolecules that exhibit antimicrobial activity against a wide range of microorganisms, including a broad group of life-threatening pathogens [35,36]. They serve as a preserved defense mechanism in various eukaryotes, such as mollusks, and have gained importance as an alternative in the treatment of infectious diseases in recent decades [37]. Since they can disrupt growth and modulate the immune system, AMPs are less likely to induce resistance in pathogens [38]. However, several AMPs are often very hemolytic and toxic to mammalian cells, making the reduction of cytotoxicity a major goal in the development of peptide drugs [39]. A few years ago, the AMP Cm-p5 (SRSELIVHQRLF; PDB: 2MP9) was discovered in the coastal tropical mollusk *Cenchritis muricatus;* it exhibits antifungal activity against the pathogen *C. auris* as well as *C. albicans* and *C. parapsilosis* [40,41,42]. Additionally, two dimeric derivatives of Cm-p5 were synthesized (parallel CysCysCm-p5 (Dimer 1) and antiparallel CysCysCm-p5 (Dimer 2)) (Figure 1C), which showed moderate antifungal activity against these yeast species, even though specific inhibition of the formation of *C. auris* biofilms was observed [11,43]. Thus, these synthetic Cm-p5 derivatives could also provide a new, specific anti-biofilm treatment in the fight against highly abundant and clinically relevant *C. albicans* and *C. parapsilosis*. To demonstrate that this activity was not limited to laboratory strains, the anti-biofilm effect was analyzed on clinical isolates of *C. albicans* collected from patients suffering from invasive infections. Such invasive isolates can be expected to be more resistant to antifungal drugs and to produce increased masses of biofilm compared to laboratory reference strains [44]. Moreover, to overcome resistance against classical antifungals, the inhibition of the formation of de novo biofilms with the Cm-p5 dimeric derivatives is highly important; therefore, the anti-biofilm activity was analyzed on two fluconazole-resistant *C. auris* strains evolved by selective pressure.

## 2. Results

### 2.1. Anti-Biofilm Activity of Cm-p5 Dimeric Derivatives against C. albicans, C. parapsilosis and Invasive Clinical Isolates

In order to optimize antifungal activity, parallel CysCysCm-p5 (Dimer 1) and antiparallel CysCysCm-p5 (Dimer 2) were generated by intermolecular disulfide bond formation, respectively [11]. The activity of these peptides against planktonic cells of both *C. albicans* and *C. parapsilosis* was only moderate but showed promising anti-biofilm properties against *C. auris* [11,43] Therefore, *C. albicans* and *C. parapsilosis* were allowed to form biofilms on microtiter plates (polystyrene surface) in the presence of increasing concentrations of both derivatives.

**Figure 2:**
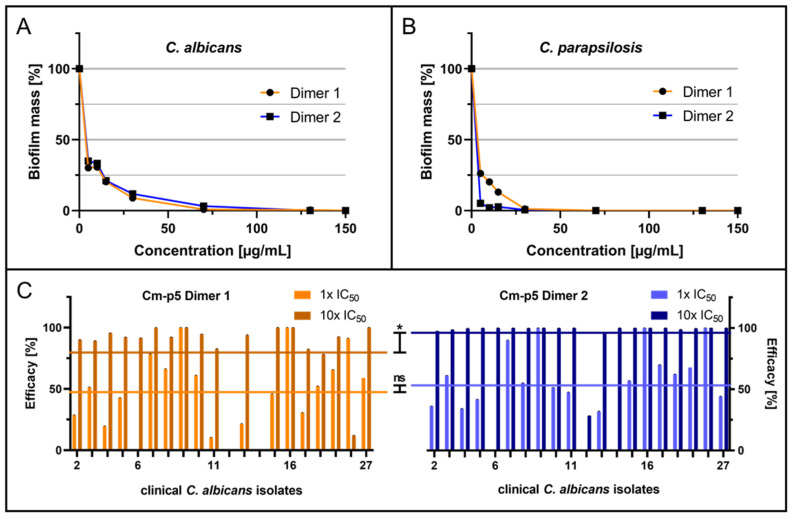
Anti-biofilm activity of Cm-p5 dimeric derivatives against *C. albicans* and *C. parapsilosis*. (**A**) Dose-dependent inhibition of *C. albicans* and (**B**) *C. parapsilosis* de novo biofilm formation by Dimer 1 or Dimer 2, quantification of biofilm mass with crystal violet after 24 h in the presence of peptides. (**C**) Evaluated effects of 6.5 µg/mL (1× IC_50_) and 65 µg/mL (10× IC_50_) for Dimer 1 (left panel) and 7.0 µg/mL (1× IC_50_) and 70 µg/mL (10× IC_50_) for Dimer 2 (right panel) on the biofilm formation of clinical *C. albicans* isolates. The average efficacies are represented by the horizontal lines, respectively. All experiments were performed in triplicate (*N* = 3), error bars indicate standard deviations. *p*-values < 0.05 were considered significant. * Denotes *p* < 0.05 and ns denotes not significant. Some standard deviations were too low to be visualized.

Using untreated yeast cells forming the reference biofilms, both dimers showed inhibitory effects in a dose-dependent manner for *C. albicans* biofilms, with semi-inhibitory concentrations (IC_50_) for biofilm inhibition of 6.5 µg/mL for Dimer 1 and IC_50_ = 7.0 µg/mL for Dimer 2 (Figure 2A). For the inhibition of *C. parapsilosis* biofilm formation, both dimers exhibited a similar reduction in biofilm mass after treatment, resulting in IC_50_ = 5.4 µg/mL for Dimer 1 and IC_50_ = 2.6 µg/mL for Dimer 2 (Figure 2B).

Considering that *Candida* strains isolated from patients suffering from infections are expected to possibly be more robust in terms of biofilm formation capability and pose high risks of developing resistances against conventional antifungal drugs, we analyzed the peptide effects against a set of invasive *C. albicans* isolates collected at Ulm University Hospital. In a previous study, these strains were introduced and characterized regarding their biofilm formation capability as well as their susceptibility to the antifungal agent fluconazole [44]. Here, only isolates that proved to form biofilms were further subjected to anti-biofilm analyses using both Dimer 1 and 2 at IC_50_ concentrations (Dimer 1: 6.5 µg/mL; Dimer 2: 7.0 µg/mL), and the respective tenfold concentrations were tested, as clinical strains were expected to be less sensitive to such peptide inhibitors. A generally moderate inhibitory effect was observed for the 1× IC_50_ concentrations, with some isolates (Dimer 1: isolates 9 and 16; Dimer 2: isolates 9, 16, and 20) being perfectly affected, but for some strains, the peptides failed completely (Dimer 1: isolate 6; Dimer 2: isolates 6, 12, and 14). Interestingly, at the tenfold peptide amounts, biofilm formation was inhibited for a considerably higher number of strains, including four individual isolates (6, 8, 11, and 13), which were previously found to be resistant against the therapeutically active dose of fluconazole of 8 µg/mL. In comparison, the overall average efficacies of Dimer 2 (1× IC_50_: 52.5%, 10× IC_50_: 95.9%) for both applied concentrations were higher than the averages for Dimer 1 (1× IC_50_: 46.2%; 10× IC_50_: 79.3%). Surprisingly, the evaluated semi-inhibitory effects were in a similar range as for the laboratory *C. albicans* ATCC 90028 as a model strain of this pathogenic yeast.

### 2.2. In Vitro Evolution of Fluconazole-Resistant C. auris 

*C. auris* as an emerging pathogenic yeast, has recently become a serious threat, causing invasive infections with high mortality rates [45]. The unambiguous identification and, in particular, the treatment of *C. auris* infections are quite challenging as resistance against several classes of antifungal drugs is widespread, and even multidrug resistance is now observed more and more frequently [46]. The triazole antifungal fluconazole is widely applied as a therapeutic in *Candida* infections; however, more than 90% of clinical *C. auris* isolates already exhibit resistance against it with minimal inhibitory concentrations ranging from 4–256 µg/mL [19]. Therefore, AMPs serve as potential candidates to overcome these resistances. In order to evaluate the effects of both Dimer 1 and Dimer 2 on *C. auris* strains, which are less susceptible to fluconazole, two strains were generated via in vitro evolution and used for further experiments.

**Figure 3:**
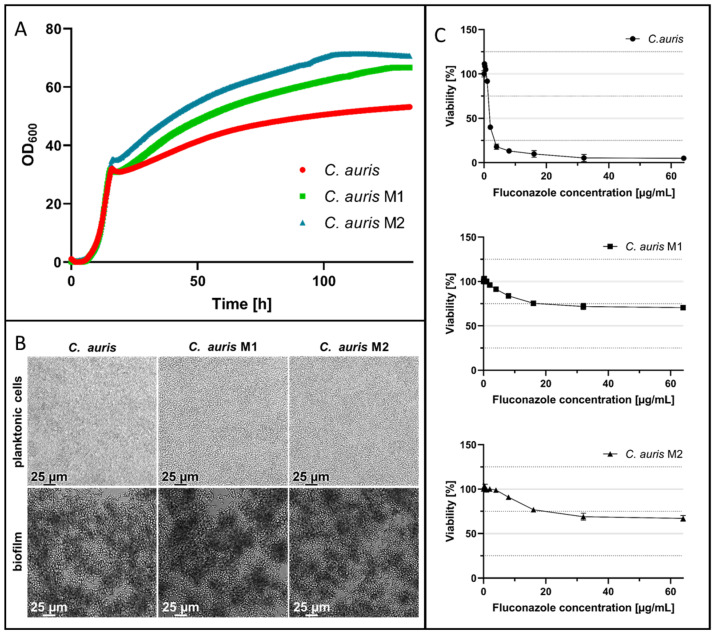
In vitro evolution of fluconazole-resistant *C. auris*. (**A**) Growth of parental *C. auris* and fluconazole-resistant *C. auris* M1 and *C. auris* M2 for 133 h at 37 °C and 180 rpm in YPD. Monitoring of the OD_600_ was performed using the CGQ by Aquila Biolabs (Baesweiler, Germany). (**B**) Microscopic analysis under transmitted light at ×20 magnitude using a Leica DMi8 coded (Leica Microsystems CMS GmbH, Wetzlar, Germany) of both planktonic cells and biofilms of parental *C. auris*, *C. auris* M1, and *C. auris* M2 after 24 h of growth. Planktonic cells were cultured with agitation at 900 rpm in RPMI-1640 medium and biofilms were allowed to form without agitation on polystyrene in RPMI-1640 medium. (**C**) Dose-dependent inhibition of parental *C. auris*, *C. auris* M1, and *C. auris* M2 viability by fluconazole; quantification of viable cells determined by resazurin reduction test after 24 h. All experiments were performed in quintuplicate (*N* = 5); error bars indicate standard deviations. Some standard deviations were too low to be visualized.

Two strains, M1 and M2, were evolved by selective pressure and exhibited similar growth patterns compared to the parental *C. auris* strain (Figure 3A) after monitoring by CGQ (Aquila Biolabs GmbH; Baesweiler, Germany). To further characterize these mutants after their evolution was complete, the morphology was inspected in comparison to the parental *C. auris* strain. The shape, size, and overall appearance were similar or identical, respectively, for planktonic cells and biofilms, which were grown for 24 h after seeding 2.5 × 10^3^ cells, as subsequently analyzed by light microscopy. The appearance of yeast cells in the planktonic phase as well as mature biofilms of *C. auris* M1 and M2 were identical to those of the parental *C. auris* cells (Figure 3B). However, the fluconazole activity was then determined for the parental *C. auris* and both of the fluconazole-evolved strains by a broth microdilution assay in accordance with CLSI’s guidelines M27-A3 [47]. *C. auris* rapidly acquired an increased fluconazole resistance in vitro, resulting in the two resistant strains M1 and M2 (Figure 3C). There, the growth of both strains in the presence of 64 µg/mL fluconazole for 24 h was reduced by only 30% for M1 and by 33% for M2 in relation to untreated yeast cells. In comparison, the viability of the parental *C. auris* strain was reduced to 5% at the highest concentration, indicating a successful evolution of resistance against this antifungal agent.

### 2.3. Anti-Biofilm Acitivity of Cm-p5 Dimeric Derivatives against Fluconazole-Resistant C. auris

An essential feature of the virulence of several pathogens such as *C. auris* is their persistence in the environment by growing biofilms on biotic and abiotic surfaces [48]. In particular, their intrinsic ability to persist on surfaces in hospital settings, including medical tubing and surgical instruments, increases the likelihood of transmission and therefore the incidence of life-threatening nosocomial infections with *Candida* spp. [49]. In an earlier study, both Cm-p5 dimers showed high anti-biofilm activity against a laboratory wildtype strain of *C. auris* [43], but activity against fluconazole-resistant strains has not yet been demonstrated.

**Figure 4:**
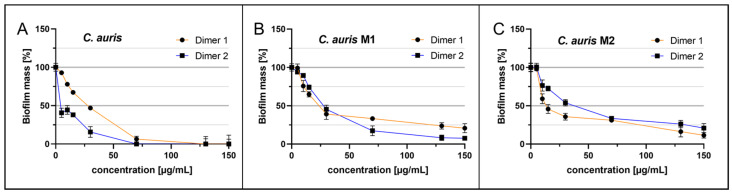
Anti-biofilm activity of Cm-p5 dimeric derivatives against *C. auris*, *C. auris* M1, and *C. auris* M2. (**A**) Dose-dependent inhibition of *C. auris*, (**B**) *C. auris* M1, and (**C**) *C. auris* M2 de novo biofilm formation by Dimer 1 or Dimer 2, quantification of biofilm mass with crystal violet after 24 h in the presence of peptides. Error bars represent standard deviations as experiments were conducted in quintuplicate (*N* = 5).

Biofilms of the parental *C. auris* strain and biofilms of fluconazole-resistant *C. auris* M1 and M2 were grown on the polystyrene surface of microtiter plates in the presence of the peptide derivatives and analyzed after 24 h of growth without agitation. Biofilm growth was considerably reduced for the original *C. auris* by Dimer 1 and Dimer 2 (Dimer 1 IC_50_ = 26.6 µg/mL; Dimer 2 IC_50_ = 9.3 µg/mL) (Figure 4A). For *C. auris* M1, 50% of biofilm growth was already reduced by Dimer 1 at 23.2 µg/mL peptide and Dimer 2 at 30.1 µg/mL (Figure 4B). Semi-inhibitory effects of Dimer 2 against *C. auris* M2 were only achieved after incubation with higher amounts of the peptide (IC_50_ = 33.3 µg/mL); however, Dimer 1 exhibited greater de novo biofilm reduction effects, as 50% of biofilms were inhibited at the addition of 16.4 µg/mL of the peptide to the cells (Figure 4C). Interestingly, in comparison, the inhibitory effect of Dimer 1 was strikingly increased against both *C. auris* mutants, with fivefold and 4.4-fold lower IC_50_ values for M1 and M2, respectively.

## 3. Discussion

Nowadays, it is known that the formation of biofilms is one of the most important virulence factors in candidiasis, with the extracellular matrix in particular contributing to antifungal resistance as antifungal drugs could be prevented from penetrating the biofilm [50]. Moreover, biofilms might be the most important way of life for microbes and especially pathogens, as their interaction with host tissues could be the decisive factor for the full accomplishment of their virulence in the event of infection [51,52,53]. One of the most common species of the *Candida* genus is the highly important pathogen *C. albicans*, which is still the most frequently isolated causative agent of candidiasis [54,55]. However, its leading role is diminishing as non-*albicans* species gain importance, particularly *C. parapsilosis* and its ability to form persistent biofilms on any abiotic or biotic surfaces such as catheters or prosthetics in medical settings [56,57,58]. Therefore, the development of specific anti-biofilm drugs is gaining importance since the treatment of biofilm-based infections is quite complicated, as cells in the biofilm state are very resilient and the limited penetration of classical antifungals causes the persistence of microbial cells even in the presence of drugs [59]. Both Cm-p5 dimeric derivatives Dimer 1 and Dimer 2 exhibit distinct activities on biofilms formed by *C. albicans* and *C. parapsilosis* in a dose-dependent fashion, as the action of these peptides completely reduced the formation of biofilm mass. Even though these findings were very auspicious, it has to be determined whether these biofilm inhibitory effects are limited to laboratory strains or if the two dimers are also effective against biofilms formed by invasive clinical isolates. These are expected to differ in their robustness, their ability to form biofilms, and, above all, their resistance to antifungals such as fluconazole. In this study, a previously described set of 20 *C. albicans* isolates, with four of them being resistant to fluconazole, has been used to determine the effectiveness of Dimer 1 and Dimer 2 on biofilm inhibition, respectively. Five isolates were already perfectly affected by the 1× IC_50_ concentrations, and both peptides failed completely in only four isolates. On average, the concentrations of Dimer 1 and Dimer 2 inhibiting biofilm formation on 50% of untreated biofilms of the laboratory control strain (IC_50_ values) were as effective against the clinical isolates, reducing biofilms by 53.8% and 45.7% (efficacies of 46.2% and 52.5% for Dimer 1 and Dimer 2). Clinical isolates must in general be expected to be resistant to drugs, as exemplified by the fluconazole-resistant strains (6, 8, 11, and 13) used here. Since it was beyond the scope of our study to characterize the individual isolates and determine exact inhibitory concentrations for each of them, we tentatively chose for the isolates the tenfold IC_50_ concentrations to estimate the highest possible efficacies of both Cm-p5 Dimers towards these strains. With this excess of peptides, biofilm formation was completely inhibited for five and seventeen strains with Dimer 1 and Dimer 2, respectively, increasing also the number of perfectly affected isolates to seventeen in total. More important was the fact that among these affected strains were those with high resistance against fluconazole, demonstrating that with the Cm-p5 dimers, biofilm-active peptides now exist as potential drug leads. The antimicrobial peptides Pom-1 and Pom-2, which were isolated from the Cuban freshwater snail *Pomacea poeyana* (Pilsbry, 1927), also exhibit pronounced anti-biofilm activities with similar semi-inhibitory concentrations for both *C. albicans* and *C. parapsilosis* [44,60,61,62]. However, with the emergence of new and aggressive pathogens such as *C. auris,* treatment of *Candida* infections is quite demanding, as resistance to all classes of available antifungals is already widespread and still increasing, especially with multidrug resistance being a true challenge [19,20]. Efficient antifungal activity against planktonic *C. auris* was previously demonstrated with Cm-p5 and even successfully used as a first-line defense against infected wounds in the form of so-called intelligent or smart wound dressings [40,41]. Cyclization of the Cm-p5 Dimers as used in our study on yeast biofilms appears to represent an important improvement of the molecule structure towards higher activity in general, since it was shown before that the antibacterial activity against various pathogenic bacteria, including *Listeria monocytogenes, Acinetobacter baumanii,* and *Enterococcus faecium* VRE, has also been improved for Cm-p5 after cyclization, and both dimeric derivatives of Cm-p5 showed overall low MIC values ranging from 12.5–25 mg/mL for Dimer 2 and MICs of 50 mg/mL for Dimer 1 [42]. Furthermore, the inhibiting activity of both Cm-p5 derivatives Dimer 1 and Dimer 2 against biofilms formed on an abiotic surface by a laboratory strain of *C. auris* was previously demonstrated [43]. However, the efficacy against *C. auris* that is resistant to the gold standard antifungal agent fluconazole has not yet been proven but is of high interest, as almost all *C. auris* strains isolated from patients already exhibit resistance to this agent [19]. To analyze the anti-biofilm activities of both Cm-p5 dimers on fluconazole-resistant strains, two mutants were classically bred by selective pressure in our laboratory, as no cases of *C. auris* were reported at Ulm University Hospital and only a few cases have occurred in Germany so far [23]. Both mutants M1 and M2 were especially susceptible to Dimer 1, which showed similar inhibitory effects on the formation of de novo biofilms compared to the parental control strain. Whereas the effectiveness of Dimer 2 against *C. auris* M1 and M2 was rather moderate after incubation with higher amounts of the peptide. Additional activities specifically against the formation of biofilm cells may be mechanistically different on the cell membranes of *Candida* spp. since classical cationic AMPs can interact with the negatively charged phospholipids of microbial cell membranes and thereby develop biocidal disruptive effects on planktonic cells by the formation of nano-scale pores [63]. One possible mode of action of Cm-p5 Dimer 1 and Dimer 2 might be the accumulation of the peptides on the cell membranes, where the homogeneity of the cell membranes certainly decreases when the peptides bind to the bilayer and thus lead to reduced membrane resistance due to a disorder in the phospholipids [64]. Peptides that are active according to the “carpet model” aggregate on the membrane immediately after addition to cells, and therefore membrane destabilization should occur. However, it has been observed that a membrane is not damaged by the peptides as it remains intact after binding [65]. If the peptides aggregate on the pathogenic cell membrane rather than destabilize them by the formation of pores, they consequently inhibit biofilm formation by disturbing cell–cell and/or cell–substratum interactions. This assumption is supported by comparing both derivatives with the original Cm-p5, which also preferentially interacts with fungal phospholipids and thus facilitates its fungistatic activity [40]. Without systematically investigating the molecular targets of Dimer 1 and Dimer 2, we can assume that microarchitectures on the cell surface and/or structures present at early stages of biofilm formation may represent a possible target for peptide activity. However, the modified dimers might have mechanistically specific activities on biofilm cells, which probably differ from the assumed membrane disruptive effects of Cm-p5, as in addition, the previously observed non-toxicity of the peptides towards macrophages and human THP-1 cells supports the theory that pore formation does not play a key role [11]. Although the significance of anti-biofilm agents was recognized in the early days of biofilm research, the development of specific therapeutics against *Candida* spp. or other pathogenic microorganisms has become a major topic only quite recently, and novel, promising molecules are still waiting to enter dedicated studies and, after their success, to enter the market. The treatment of biofilm-based infections still remains a challenge, and we believe that with the peptide dimers presented here, a new set of molecules has been added to the portfolio of promising candidates that are worth serving as lead structures to develop potent new anti-biofilm drugs against pathogenic *Candida* yeasts.

## 4. Materials and Methods

### 4.1. Materials

Acetic acid, agar-agar, crystal violet, glucose, 3-(N-morpholino)propanesulfonic acid (MOPS), peptone, and yeast extract were purchased from Carl Roth GmbH (Karlsruhe, Germany), and RPMI-1640 medium supplemented with L-glutamine was obtained from Thermo Fisher Scientific (Waltham, MA, USA). Fluconazole was ordered from Merck KGaA (Darmstadt, Germany), and resazurin sodium salt was purchased from Sigma-Aldrich Chemie GmbH (Steinheim, Germany).

### 4.2. Microorganism Strains and Growth Conditions

*C. albicans* (ATCC 90028) and *C. parapsilosis* (ATCC 22019) were obtained from the Laboratory of Medical Mycology, IPK, and *C. auris* (DSMZ-No. 21092) was purchased from DSMZ. Clinical *C. albicans* isolates were provided from the patient samples sent to the Microbiology Department for diagnostic purposes. Strains were collected anonymously, and it is therefore not possible to assign the strains to patients. The accreditation number of the Microbiology Department is DIN EN ISO15189:2014 (DAkks). All isolates were grown on Sabouraud dextrose agar (40 g/L glucose, 10 g/L peptone, 20 g/L agar, pH 5.6) at 37 °C for 16 h. For suspension cultures, 10 mL of RPMI-1640 medium supplemented with L-glutamine in a 100 mL Erlenmeyer flask was inoculated, separately, with a single colony for each strain and grown at 37 °C with orbital shaking at 150 rpm for 16 h.

### 4.3. In Vitro Evolution of Fluconazole-Resistant C. auris Strains

In order to generate *C. auris* strains that are resistant to the common antifungal agent fluconazole, *C. auris* was grown on yeast extract peptone dextrose (YPD) agar (1% *w*/*v* yeast extract, 2% *w*/*v* peptone, 2% *w*/*v* glucose, 1.5% agar-agar) supplemented with 8, 16, or 32 µg/mL fluconazole. For suspension cultures, 10 mL of YPD medium supplemented with 8, 16, or 32 µg/mL fluconazole in a 100 mL Erlenmeyer flask was inoculated with a single colony and grown at 37 °C with orbital shaking at 150 rpm for 16 h. To obtain fluconazole-evolved *C. auris* strains, the yeast was cultured in YPD supplemented with 8 µg/mL fluconazole, and afterwards cultures were plated on YPD agar containing the same concentration of fluconazole. Then, two individual colonies were picked for further characterization. Two fluconazole-evolved strains, M1 and M2, were subsequently further passaged in YPD supplemented with 16 and 32 µg/mL of fluconazole, respectively. Both cultures were then again plated on YPD agar containing the same concentration of fluconazole, and again individual colonies were picked for further characterization. For further experiments, these resistant strains were grown on Sabouraud dextrose agar at 37 °C for 16 h and for suspension cultures, 10 mL of RPMI-1640 medium supplemented with L-glutamine in a 100 mL Erlenmeyer flask was inoculated with a single colony and grown at 37 °C with orbital shaking at 150 rpm for 16 h. Growth monitoring of *C. auris*, *C. auris* M1, and *C. auris* M2 was performed according to Bruder et al. using the Cell Growth Quantifier (CGQ) by Aquila Biolabs GmbH (Baesweiler, Germany) [66]. In brief, 100 mL of YPD-medium was inoculated with each *C. auris* strain to an OD_600_ of 0.3, and the growth was monitored for 133 h at 37 °C with shaking at 180 rpm, respectively. Microscopic evaluation of mature biofilms and planktonic cells was carried out using a Leica DMi8 coded (Leica Microsystems CMS GmbH, Wetzlar, Germany) at ×20 magnitude.

### 4.4. Viability Tests and Quantification

For *C. auris* and *C. auris* fluconazole-resistant mutants (M1 and M2), the viability of the yeasts in the presence of different amounts of fluconazole was determined according to the “Clinical and Laboratory Standards Institute” (CLSI) guidelines M27-A3 broth microdilution assay [47]. In brief, 2.5 × 10^3^ yeast cells were seeded in 200 μL of RPMI-1640 medium supplemented with L-glutamine in microtiter plates (flat-bottomed, 96-well, polystyrene) (Sarstedt AG & Co., KG, Nümbrecht, Germany) and incubated at 37 °C for 24 h with agitation at 900 rpm on an Eppendorf shaker. The cell viability was quantified by a resazurin assay, according to Patricia Bi Fai et al. [67]. The cells were incubated with 20 μL of resazurin at a concentration of 0.15 mg/mL for 2 h. Living cells reduce resazurin to fluorescent resorufin through the production of NADPH. The amount of produced resorufin was analyzed by fluorescence measurements at an excitation wavelength of 535 nm and an emission wavelength of 595 nm with a Tecan Infinite F200 microplate reader to quantify the viability.

### 4.5. Biofilm Formation and Quantification

Biofilms were formed and analyzed in triplicate, as described previously [68,69,70]. In brief, 2.5 × 10^3^ yeast cells were seeded in 200 µL of RPMI-1640 medium supplemented with L-glutamine in a microtiter plate (flat-bottomed, 96-well polystyrene) (Sarstedt AG & Co., KG, Nümbrecht, Germany) and incubated at 37 °C without agitation for 24 h. The effect of the different Cm-p5 dimeric derivatives on biofilm formation was tested at different concentrations of the peptides. Quantification of the biofilm was done by a crystal violet assay, which was originally developed for bacteria by George O’Toole [70,71], and is also widely used for *Candida* biofilms [68,69,72,73]. Planktonic cells were removed, and the mature biofilms were washed twice with 200 µL of water. Subsequently, biofilms were stained with 200 µL of a 0.1% (*w*/*v*) crystal violet solution for 15 min. Then the supernatant was removed, and the biofilms were washed twice with 200 µL water in order to remove excess crystal violet. The stained biofilms were air dried for 24 h at 25 °C and finally destained using 200 µL of 30% acetic acid for 15 min at 25 °C. The supernatant was transferred to a new 96-well plate, and the absorbance at 560 nm was measured using a Tecan Infinite F200 microplate reader to quantify the biofilm biomass. The dose-response curves were fitted by spline fitting with interpolated x-values using GraphPad PRISM8 (GraphPad Software, Inc., San Diego, CA, USA). The semi-inhibitory concentration of biofilm formation (IC_50_) represents the point at which the biofilm mass is reduced to 50% compared to the biofilm mass of the untreated control. With Student’s *t*-test, the statistical significance was tested. *p*-values < 0.05 were considered significant; * denotes *p* < 0.05, and ns denotes not significant. Standard deviations were represented as error bars.

## 5. Conclusions

Anti-biofilm agents are of increasing interest, especially in the age of emerging resistance development, not only to prevent biofilm formation but also to possibly regain the activity of standard antimicrobial therapeutics such as fluconazole. Here we have demonstrated that dimeric derivatives of the already described AMP Cm-p5 can be efficiently used to inhibit the formation of de novo biofilms grown by the highly common pathogenic yeasts *C. albicans* and *C. parapsilosis*. Moreover, this anti-biofilm effect was not limited to laboratory strains; the peptides were also effective against a set of resistant invasive clinical isolates of the *C. albicans* species. Additionally, two fluconazole-resistant strains of the relatively new but problematic pathogen *C. auris* were evolved by selective pressure, and anti-biofilm activity was also observed for both peptides.

## Data Availability

Not applicable.
